# Integration of Augmented Reality Into Glioma Resection Surgery: A Case Report

**DOI:** 10.7759/cureus.53573

**Published:** 2024-02-04

**Authors:** Rachel Hunt, Lisa Scarpace, Jack Rock

**Affiliations:** 1 Neurosurgery, Henry Ford Health System, Detroit, USA

**Keywords:** glioblastoma, medical device, cranial neurosurgery, mixed reality, augmented reality

## Abstract

Augmented reality (AR) is an exciting technology that has garnered considerable attention in the field of neurosurgery. Despite this, clinical use of this technology is still in its infancy. An area of great potential for this technology is the ability to display 3D anatomy overlaid with the patient to assist with presurgical and intraoperative decision-making. A 39-year-old woman presented with headaches and was experiencing what was described as a whooshing sound. MRI revealed the presence of a large left frontal mass involving the genu of the corpus callosum, with heterogeneous enhancement and central hemorrhagic necrosis, confirmed to be a glioma. She underwent a craniotomy with intraoperative MRI for resection. An augmented reality system was used to superimpose 3D holographic anatomy onto the patient's head for surgical planning. This report highlights a new AR technology and its immediate application to cranial neurosurgery. It is critical to document new uses of this technology as the field continues to integrate AR as well as other next-generation technologies into practice.

## Introduction

Glioblastoma multiforme (GBM) stands as one of the most aggressive and challenging brain tumors to treat, necessitating innovative solutions to enhance surgical precision and patient outcomes [1,2]. In recent years, the integration of augmented reality (AR) technology into the field of neurosurgery has emerged as a transformative tool. The conventional approaches to glioblastoma resection have long relied on neuroimaging and surgical navigation systems. However, the limitations of these methods in providing real-time, three-dimensional visualization of the tumor and surrounding structures have spurred the exploration of AR. By seamlessly merging digital information with the surgeon's view of the patient's anatomy, AR enhances spatial orientation and understanding, and can provide new perspective when surgically planning a case [3-8]. This case report describes the integration of an augmented reality system into the presurgical workflow of a GBM resection surgery and provides a glimpse into the potential applications of this emerging technology. We explore the advantages and limitations of AR with the goal of drawing greater awareness to this technology.

## Case presentation

**Figure 1:**
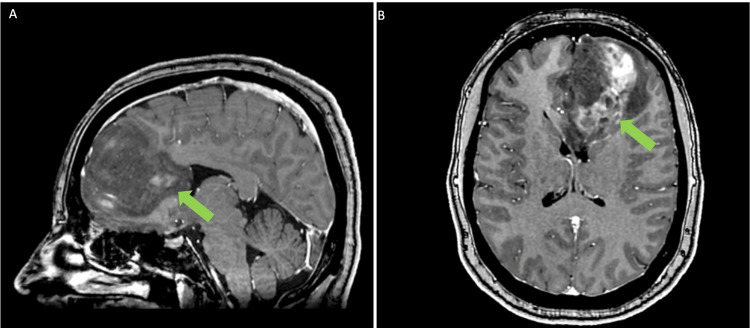
Preoperative Imaging A) Sagittal and B) Axial view of patient preoperative MRI. Location of frontal glioma indicated by green arrow.

A 39-year-old woman presented with headaches and was experiencing what was described as a whooshing sound. MRI revealed the presence of a large left frontal mass involving the genu of the corpus callosum, with heterogeneous enhancement and central hemorrhagic necrosis, confirmed to be a glioma (Figure 1). She underwent a left front craniotomy with brainlab navigation and intraoperative MRI for resection.

Augmented reality technology platform

An AR system was incorporated into the surgical workflow. The software (Hoth Intelligence, Philadelphia, PA, USA) functions on the Microsoft Hololens 2 head-mounted display (Redmond, WA, USA). The Microsoft Hololens 2 is an untethered optical see-through head-mounted display that displays digital content (i.e., holograms, images, screens) onto the users’ real-world field of view [9,10].

Mixed reality presurgical planning

**Figure 2:**
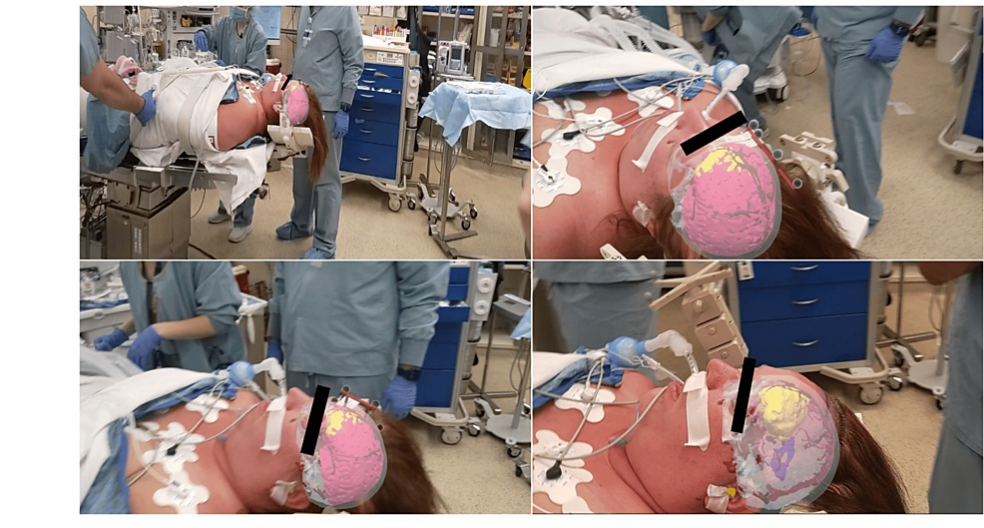
Augmented Reality Visualization User’s view through the augmented reality (AR) headset displaying a 3D model overlaid onto the patient's head. User can move around the patient and view the 3D model from different perspectives.

The AR technology used for this case overlays 3D models of the patient’s anatomy onto the head via a fiducialess registration process. When the user looks at the patient’s face, the model is registered and displayed on the patient’s head. The patient's model consisted of six layers including face (gray), ventricles (blue), vasculature (red), brain (pink), skull (white) and tumor (yellow). The addition of an infrared (IR) sphere array, while not required for registration, allows the surgeon to manipulate and adjust the patient's head prior to pinning and visualize how the 3D digital anatomy moves with patient head adjustments. Prior to pinning the patient a 3D model was registered onto the patient’s head in the operating room (Figure 2). Once registered onto the head, the surgeons were able to move the head and visualize the underlying lesion in 3D. Following patient pinning, while wearing the AR headset, the surgeon visualized the registered anatomy which assisted with determining surgical approach and craniotomy location prior to setting up neuronavigation.

Operative and postoperative course

**Figure 3:**
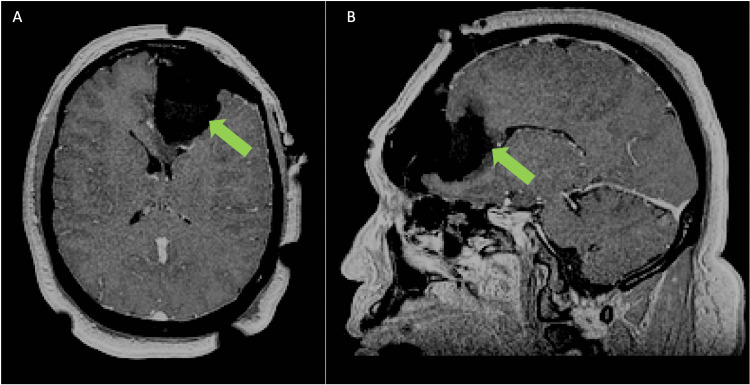
Postoperative Imaging A) Axial and B) Sagittal view of patient postoperative MRI. Green arrows indicates location of resected glioma.

The patient was supine. Augmented reality was used to visualize the patient's registered 3D anatomy prior to and after pinning. Following AR planning and brainlab neuronavigation setup, the patient was prepped and draped and a craniotomy was performed to remove the left frontal bone plate. During debulking of the tumor a border laterally, posteriorly, anteriorly and inferiorly was established after which the tumor was removed from the corpus callosum under and posterior to the anterior cerebral artery (A2) vessels. After visual and 2D navigation demonstrated gross total resection the patient was prepared for intra-operative MRI (iMRI). After this was done remaining tumor was noted and removed. Based on postoperative MRI, a gross total resection was achieved (Figure 3).

## Discussion

While interest in AR for neurosurgical use is rapidly growing, at this early junction, it is imperative to document the various uses of this technology if it is to ultimately become standard of care [4-6]. Here we present an early use of AR for assisting in the surgical planning for a glioma resection. We describe a new AR system that can be seamlessly integrated into the clinical workflow. In this case, the surgeon was able to visualize the location of underlying anatomic structures-tumor, brain tissue, vasculature, skull-registered onto the patient’s head for the purposes of refining a surgical plan.

In order for AR to become readily adopted into the field of neurosurgery, it is critical that the technology can be integrated into clinical care with minimal disruption or delay of the surgical workflow. As such the system must be fast, user-friendly, and have a small operating room footprint. The system described allows registration of patient imaging without the need for fiducials. The surgeon visualizes the patient's head while wearing the headset, and a series of algorithms overlay the 3D anatomic model onto the patient’s head. Registration can be completed in ~15 seconds, and the hardware required is only an AR headset. There are no other hardware components (i.e. computers, external cameras) that require space in the operating room.

There is a growing literature describing the use of AR for neurosurgical cases [7,11,12]. In most reports, AR is used to display and study 3D patient anatomy, however the models aren’t registered onto the patient’s head [8,11,12]. While this is valuable for planning complex cranial cases, overlaying the 3D model onto the patient’s head was helpful for confirming proper patient positioning and craniotomy location. This is certainly valuable from a clinical standpoint but has tremendous educational value as well for trainees learning surgical approaches. This report describes a basic yet important application of AR for refining a surgical plan. Determining the surgical plan prior to the case is fundamental however, the ability to enhance, improve, or confirm the plan with 3D anatomy visualized over the patient's head was of immense value. There are however numerous aspects of this technology that warrant further exploration. The AR system was used in conjunction with neuronavigation. While the position of the 3D tumor appeared correctly overlaid onto the head in the proper location and orientation, and aligned nicely with the planned craniotomy, we did not formally measure the concordance between the AR system and the neuronavigation system. This will be critical to assess in future work. It remains to be seen whether AR will replace neuronavigation or rather synergize with it. There are certainly applications when image guidance is unavailable (i.e. drain, shunt, hematoma evacuation) where a system like this can lead to greater safety and precision. The AR system was not used intraoperatively in this case, however, we think it might have been easier to appreciate the full extent of tumor removal before intraoperative MRI by visualizing the 3D tumor model post-resection. We plan to assess the intraoperative value of the AR visualization in future cases. Continued use and testing of this technology including accuracy, speed, and patient outcomes will reveal new opportunities and advantages of AR for neurosurgical care.

## Conclusions

This case report demonstrates the institutions’ first experience of incorporating AR into the surgical planning of a glioma resection case. It is our hope that this draws greater interest and introduces the potential of this exciting type of technology. While the benefits of this technology are becoming increasingly apparent, for future studies it will be critical to formally evaluate the advantages of AR. Therefore studies addressing time and cost savings, clinical outcomes, and accuracy are critical.
